# Stimulus-specific adaptation in a recurrent network model of primary auditory cortex

**DOI:** 10.1371/journal.pcbi.1005437

**Published:** 2017-03-13

**Authors:** Tohar S. Yarden, Israel Nelken

**Affiliations:** Department of Neurobiology, the Alexander Silberman Institute of Life Sciences and the Edmond and Lily Safra Center for Brain Sciences, Hebrew University, Jerusalem, Israel; Research Center Jülich, GERMANY

## Abstract

Stimulus-specific adaptation (SSA) occurs when neurons decrease their responses to frequently-presented (standard) stimuli but not, or not as much, to other, rare (deviant) stimuli. SSA is present in all mammalian species in which it has been tested as well as in birds. SSA confers short-term memory to neuronal responses, and may lie upstream of the generation of mismatch negativity (MMN), an important human event-related potential. Previously published models of SSA mostly rely on synaptic depression of the feedforward, thalamocortical input. Here we study SSA in a recurrent neural network model of primary auditory cortex. When the recurrent, intracortical synapses display synaptic depression, the network generates population spikes (PSs). SSA occurs in this network when deviants elicit a PS but standards do not, and we demarcate the regions in parameter space that allow SSA. While SSA based on PSs does not require feedforward depression, we identify feedforward depression as a mechanism for expanding the range of parameters that support SSA. We provide predictions for experiments that could help differentiate between SSA due to synaptic depression of feedforward connections and SSA due to synaptic depression of recurrent connections. Similar to experimental data, the magnitude of SSA in the model depends on the frequency difference between deviant and standard, probability of the deviant, inter-stimulus interval and input amplitude. In contrast to models based on feedforward depression, our model shows true deviance sensitivity as found in experiments.

## Author summary

We present a possible mechanism for the way auditory cortex emphasizes stimuli that are deviant within a regular, repetitive sequence. This enhancement is strong and widespread in auditory cortex, but not in its major thalamic input, the ventral division of the medial geniculate body. In contrast with previous models, which are based on depression of the synapses that convey the input to the cortex, here the network structure and the known dynamics of intracortical synapses play a key role. The model accounts better than previous models for available experimental data, and provides testable predictions that differentiate it from feedforward models. It is a useful starting point for studying the circuit mechanisms that underlie cortical responses to unexpected stimuli.

## Introduction

Stimulus-specific adaptation (SSA) is the decrease in responses to a repeating stimulus (standard) that does not generalize to another, rarely-occurring stimulus (deviant). SSA is a robust and widespread finding in the auditory system. It has been demonstrated in single neurons in primary auditory cortex (A1) of anesthetized cats [1], and in single-unit, multi-unit and local field potential (LFP) recordings in A1 of awake [2] and anesthetized rats [3,4]. Although SSA is present in the inferior colliculus [5–7] and the auditory thalamus [8–11], it is mostly confined to the non-lemniscal pathway [8,9] and is thus likely generated *de novo* in A1, whose major thalamic input is from the lemniscal part of the medial geniculate body. SSA in A1 has true deviance sensitivity: The response to a rare stimulus presented within a sequence composed mostly of the same standard tone (which violates the expectation for yet another repeat of the standard) is larger than the response to the same rare stimulus when presented within a sequence composed of many different tones (that doesn't generate strong expectations for any stimulus) although the level of sensory adaptation in the multi-tone sequence may be lower [4,12,13]. SSA is therefore an appealing test case for linking high-level concepts such as deviance sensitivity with mechanistic models.

SSA shares many similarities with mismatch negativity (MMN) [14], an event-related potential evoked by deviant stimuli that has been investigated extensively in humans [15]. While it is clear that SSA is not the direct neuronal correlate of MMN [16,17], it may be one of the stages leading to MMN. In fact, human midlatency potentials correspond better with SSA temporally. Like SSA, midlatency potentials show deviance sensitivity [18]. It is therefore tempting to hypothesize that cortical SSA is the direct neural correlate of deviance sensitivity in midlatency potentials.

There exist a number of models for deviance sensitivity, mostly in the context of the MMN. While some models are based on sensory adaptation [19,20], others postulate an explicit prediction mechanism [21]. These models, however, are not very realistic at the single-neuron and local network level. In models based on sensory adaptation, changes in membrane excitability, which are a prominent mechanism of neural adaptation [22,23], cannot easily explain SSA as they would affect similarly the responses to all stimuli. Therefore, SSA models have been primarily based on synaptic adaptation, which can be stimulus-specific.

SSA may arise from synaptic depression in the thalamocortical (ThC) input to A1, as studied e.g. by Lee and Sherman [24]. Indeed, computational models based on purely feed-forward connectivity show SSA [4,25,26]. However, these models provide predictions that are inconsistent with some of the experimental data [4,12]. Here we study an alternative mechanism: SSA may arise from the heavily recurrent cortical connectivity and the depression of the local, intracortical synapses in A1 [27]. We use a modified version of a recurrent neural network model with synaptic depression [28], which reproduced important properties of A1 responses, including frequency tuning, forward masking, and lateral inhibition. An important feature of this model is the generation of synchronized firing events called population spikes (PSs). The existence of PSs in A1 has support from intracellular [29,30] and multi-unit recordings [31–33], and from network [34] and single-cell calcium imaging studies [35]. We study the basic properties of SSA and its robustness using both simulations and mathematical analysis, and provide predictions for experimental testing of the role of intracortical synaptic depression in cortical SSA.

## Results

**Fig 1:**
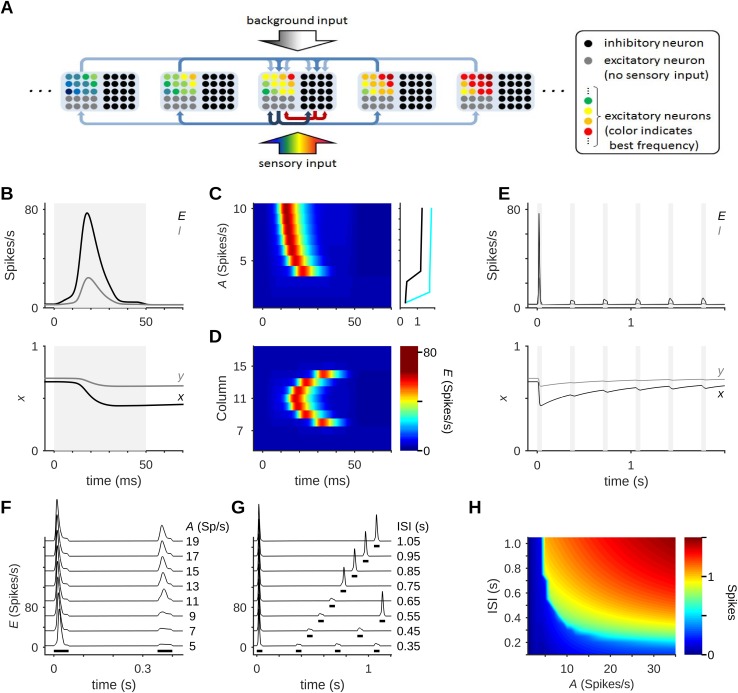
Architecture and activity of the model network. (A) The primary auditory cortex is represented by a recurrent neural network divided into cortical columns. Columns are arranged tonotopically by their best frequencies (BFs), but the BFs of neurons within each column show some variability (BFs are represented by color). Arrows illustrate the connectivity of one column. (B) Upon sensory stimulation a column may generate a population spike (PS), shown here as a transient increase in the column’s mean firing-rate, *E* (top). The inhibitory population (mean firing-rate, *I*) lags behind the excitatory activity. A PS depletes the synaptic resources, which recover gradually (bottom; *x* is the mean fraction of available resources in excitatory synapses, *y* in inhibitory synapses). Stimulus duration shown in gray. (C) The threshold for PS generation. Left: Time course of responses for stimuli of varying amplitude. Response strength is displayed using a color scale. Right: Spike count (integrated from stimulus onset until 45 ms following offset; see Methods) as a function of input amplitude, with (black) and without (cyan) feedback inhibition. (D) A PS that is initiated in one column (here, column 11) can propagate across the network. The color scales of (C) and (D) are identical. Stimulus duration in (C) and (D) is the same as in (B). (E) A low level of synaptic resources (x) may prevent subsequent stimuli from evoking a PS. Stimulus presentations are shown in gray. (F,G) Refractoriness of PS generation. Following a PS, a stimulus has to be stronger (F) or presented at a longer latency (G) in order to evoke a PS. Stimuli marked by black bars under the traces. In (F), the first stimulus has *A* = 5 Spikes/s, and values next to the traces specify the amplitude of the second stimulus. In (G), all stimuli are of the same amplitude, *A* = 5 Spikes/s. (H) The spike count for the second stimulus. The first stimulus was always presented with *A* = 5 Spikes/s. The second stimulus varied in amplitude (abscissa) and ISI (ordinate).

We model primary auditory cortex (A1) as a neural network with multiple cortical columns (Fig 1A). The crucial property of our model is the use of synaptic depression in all of the excitatory connections, both intracortical and thalamocortical. A similar model has been used successfully to model many properties of auditory cortex [28]. Here we show that this model also exhibits stimulus-specific adaptation (SSA). Our model network often responds to sensory stimulation by generating population spikes (PSs). PSs are events in which a large number of the neurons in a column fire a spike within a few milliseconds, and are characteristic of recurrent networks with synaptic depression [28,36,37]. A PS is generated when there are enough synaptic resources in the network to allow a spike in one neuron to evoke spikes in its post-synaptic targets. It is a regenerative event in which a large fraction of the neurons in the network is rapidly recruited to a brief period of general firing. In a rate model such as the one studied here, PSs are expressed as transient increases in the mean firing-rate within a column (Fig 1B, top). The expected number of spikes fired by each individual neuron may not be large–in fact, it is typically about 1 spike for each PS in the simulations shown here. However, the fact that many neurons fire this spike within a short period of time causes a depletion of the synaptic resources (denoted later by *x*) within the column, followed by a gradual recovery with a time-constant *τ*_rec_ (Fig 1B, bottom). Consistent with their regenerative nature, PSs are characterized by a sharp threshold (Fig 1C, left). Below threshold, PSs are not evoked. Above threshold, PSs are always evoked, although stronger input amplitude produces PSs with shorter latency and somewhat higher peak firing- rate.

Each column in our model consists of an excitatory and an inhibitory population. The inhibitory neurons do not get a direct thalamic input, contrary to the known physiology. The reason is technical: the behavior of the model does not change much when adding a low amount of direct thalamic input to the inhibitory neurons. However, when adding a substantial amount of thalamic input, as found in experiments, the sensory responses are completely quenched, because rate models cannot easily produce the lag between excitation and inhibition consequent to the additional synaptic delay between inhibitory and excitatory neurons [38]. In order to produce this delay, we kept only the feedback inhibition. Indeed, in this configuration, the firing rate of the inhibitory population, *I*, lags behind the excitatory population’s firing rate, *E* (Fig 1B, and cf. also [28]) and acts to both restrain the amplitude of the PS and increase its threshold (Fig 1C, right).

Importantly, once PSs are initiated, they can propagate from one column to the next (Fig 1D). The response latency in the next columns can be tens of milliseconds longer than in the column where the PS originates, as has also been observed both in this model [28] and experimentally [39]. PSs propagate over a substantial part of the model network. The extent of the propagation is limited by the extent of the sensory input, which has to be large enough in order to prime a column to allow PS propagation into it. Specifically, each column in our model corresponds to about 1/3 octave along the tonotopic gradient of rat A1 and sensory input consisting of a single tone activates a region covering about 2 octaves, in line with typical tuning-curve widths in rat A1 at suprathreshold levels (see Materials and Methods; cf. [40]). Under these conditions, the evoked PS typically propagated into 3 columns on each side of the column where it initiated, making the population response more or less co-localized with the thalamic input (as found experimentally e.g. by Li et al. [41]).

Because of synaptic depletion, the recurrent connections in the column are effectively weaker following a PS. This may prevent subsequent stimuli from generating another PS (cf. Fig 1E). The adaptation of columns in our model is fast, with the population response failing to generate another PS already upon the second stimulus. Examples of fast adaptation in *in-vivo* recordings may be found in [42,43]. Depression in the recurrent connections is thus the basic mechanism of adaptation to repetitive stimulation in this model. Following a PS, the depressed column enters an essentially refractory period, during which the threshold for a subsequent PS is increased (Fig 1F), decreasing slowly back to normal (Fig 1G). During a refractory period, even when a stimulus evokes a response, the PSs are smaller in amplitude than the initial PS. Fig 1H shows how the amplitude of the response to the second stimulus (in spike counts) varies with the input amplitude of the second stimulus and the time since the previous stimulation. Responses qualify as a second PS if their spike count is close to or higher than 1.

It is important to bear in mind that sparse, asynchronous firing can still occur during the period following a PS. Evoked activity is also possible during this period, but the few neurons activated will usually not be able to recruit the network for another PS. This stems from insufficient synaptic resources, as even a small depletion can greatly affect the ability of the network to generate a PS.

### SSA results from depression of the recurrent synapses

**Fig 2:**
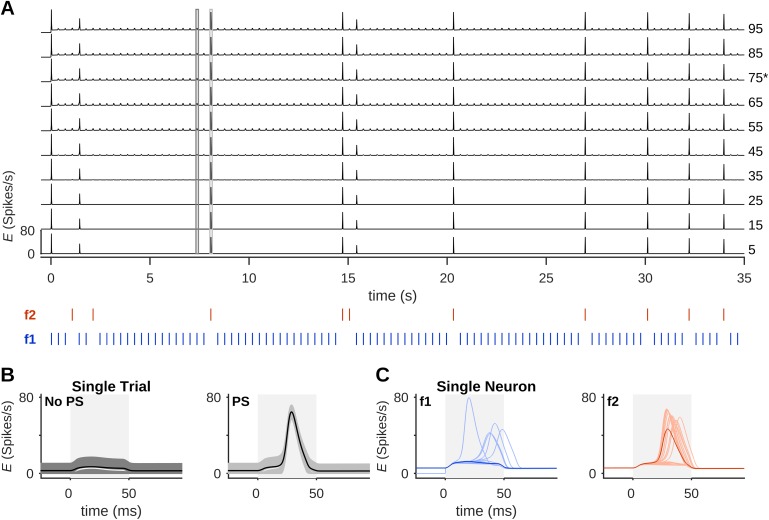
Dynamics of single-neuron and population activity. (A) The firing-rate, *E*, of selected neurons in column 11 in response to an oddball protocol (stimuli shown as blue and red bars under the plot). PS responses were usually evoked by deviant stimuli (red) and only rarely by standard stimuli (blue). (B) Single neurons were highly correlated in their single-trial responses, showing either a small transient increase in firing-rate (left) or a sharp concerted increase in firing-rate, which marks a PS (right). (C) All responses of a single neuron (no. 75, marked with an asterisk in (A)) to the standard condition (left, light blue curves) and to the deviant condition (right, light red) during 5 blocks similar to the one shown in (A). Blue and red curves show the average response of this neuron in each condition. In all blocks, input amplitude (*A*) = 5 spikes/s and inter-stimulus interval (ISI) = 350 ms.

SSA is commonly demonstrated using two tones that evoke similar responses in the recording site [1] and presenting them repetitively within an oddball protocol, where one tone occurs with high probability (standard) and the other occurs with low probability (deviant; cf. Fig 2A). Hence, we analyzed SSA in the column midway between the “standard column” and the “deviant column” (the columns whose best frequencies were the standard and deviant frequencies, respectively), referred to as the “middle column”. As evident from Fig 2A, SSA occurred in the model because standard tones often could not produce PSs in the middle column, yet the deviant tones could.

SSA in this model looks somewhat different when considered from the point of view of the single neuron (averaging many single-trial responses) and from the point of view of the single-trial network activity (averaging across many neurons), a dissociation that has been studied experimentally in auditory cortex (e.g. [32]). Fig 2A shows the firing-rates of selected neurons in a single block of the oddball protocol consisting of 100 stimuli. In each single trial, neurons showed one of two response types. The first was a small transient increase in firing-rate (Fig 2B, left, showing the responses of all neurons in trial no. 22). The other is the signature of a PS, a concerted sharp increase in firing-rate (Fig 2B, right, showing the responses of all neurons to trial no. 24) during which each neuron fired on average about one spike. Population spikes occurred mostly (but not exclusively) in response to deviant tones, as will be discussed in more detail below.

The responses of single neurons across many presentations of the same stimulus could show substantial variability. The responses of a neuron to the standard tones (e.g. neuron no. 75. Fig 2D, top, shows responses from 500 trials, including those shown in Fig 2A) mostly consisted of small increases in firing-rate but included also a few large responses that occurred when standards did evoke a PS (light blue traces in Fig 2C, left; average response in blue). The responses of the same neuron to the deviant tones were mostly large, although they also included a few small responses, corresponding to failures of a deviant to evoke a PS (e.g. trial no. 4 in Fig 2A; Fig 2C, right, light red traces; average response in red).

In conclusion, sensory responses may occur in a single neuron whether or not a population spike occurred. The average response of a neuron to both standards and deviants is composed of trials with PSs, with a large and consistent response across neurons, and trials with no PSs. The difference between standard and deviant responses has to do with the probability of such events: PSs are likely to occur in response to deviant tones, with a few failures. On the other hand, standard responses show a high probability of failures, but the successful PS responses to the standard are similar in their magnitude to those evoked by the deviant. This situation is also found in experimental results [12,43].

**Fig 3:**
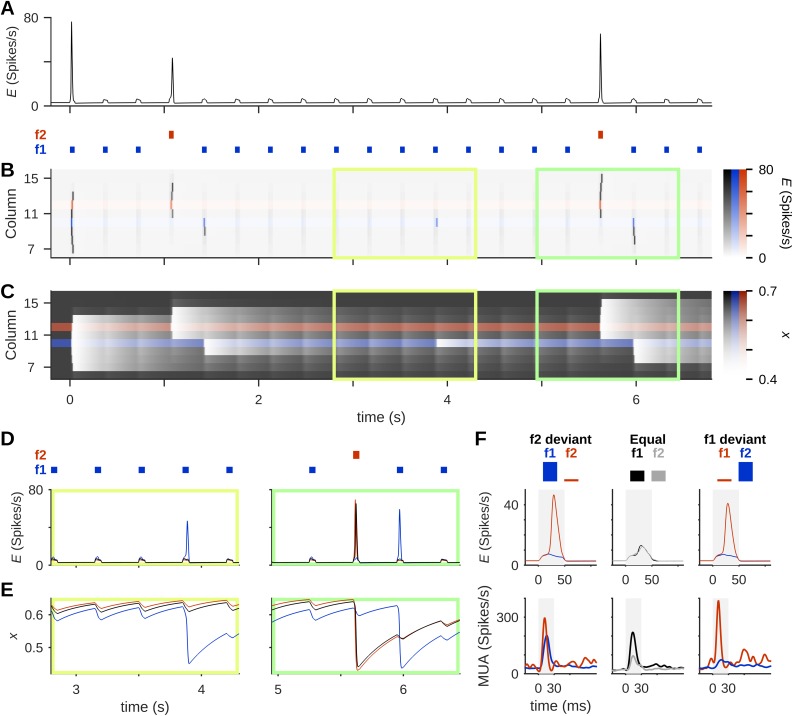
Stimulus-specific adaptation (SSA) in the model network. Presenting the network with two tones with different probabilities of occurrence produced SSA in a column that responds to both tones. (A) The mean firing-rate, *E*, in column 11 in response to an oddball protocol (stimuli marked as blue and red bars under the plot). Upon presentation of two tones, f1 = 10 and f2 = 12 (tone “frequency” = index of the column most sensitive to the tone), the middle column showed PS responses only to the deviant. (B) Mean firing-rates across multiple columns. PSs were sometimes generated in column 10 (the standard column; blue), but apart from the response to the first tone in the protocol, only PSs from column 12 (deviant column; red) were strong enough to propagate into column 11. (C) The time-course of synaptic resources, *x*, across the network shows the long-term effects of PS events: the strong resource depletion left in their wake. In (B) and (C), the standard and deviant columns are shown with different color maps from the rest of the network (black), in order to highlight their locations and roles in the network activity. (D) and (E) Close-ups on the mean firing-rate (D) and mean synaptic resources (E) in the deviant (red), middle (black) and standard (blue) columns for the periods marked with the corresponding colored rectangles in B & C, showing that PSs initiated in the deviant column were able to invade the middle column but PSs initiated in the standard column mostly failed to do so. (F) Top: Traces of average responses in column 11 to both frequencies in the two oddball conditions (Deviant f2, Deviant f1) as well as in the Equal condition. The strength of response to each tone depended on its probability of occurrence (represented in the bar graphs above the traces). Bottom: An example for multi-unit activity (MUA) responses from one site in rat auditory cortex to two frequencies (f1 = 13.3, kHz f2 = 19.2 kHz) in the two oddball conditions and the Equal condition. Data replotted from Taaseh et al. [4]. Stimulus duration is marked in gray. In all simulation protocols, input amplitude (*A*) = 5 spikes/s and inter-stimulus interval (ISI) = 350 ms.

To illustrate the mechanisms of SSA in the model, Fig 3A–3C show the firing-rates and time-course of synaptic resources not only in the middle column but also in its neighbors. PSs left a strong depletion of resources in their wake (Fig 3C). Both the deviant and standard tones initiated PSs in their respective columns. However, standard tones initiated PSs much less often in the standard column, and even those that were initiated mostly failed to propagate into the middle column. Importantly, as discussed above, even when a stimulus failed to evoke a PS, sensory responses to that stimulus still occurred at least in some of the neurons in the column.

PSs initiated in the standard column were of two types. Occasionally a PS could be evoked in the standard column during a train of standard tones. This occurred due to gradual recovery of resources from one tone to the next (Fig 3D and 3E, left panels). Such PSs failed to propagate outside the standard column because of their small amplitude. The other type consisted of PSs evoked in the standard column by the first standard following a deviant (Fig 3D, right panel). These PSs occurred because the deviant tone did not deplete the resources in the standard column as much as another standard tone would have done (Fig 3E, right panel). This was primarily due to the adaptation in the standard column, which prevented the PS evoked by the deviant from propagating into it, resulting in little depletion of resources during the presentation of the deviant. In consequence, following the presentation of a deviant, the available resources in the standard column were higher than following the presentation of a standard tone, sometimes allowing PS generation in response to the next standard tone. However, in these circumstances the propagation of PSs from the standard column into the middle column generally failed, due to depletion of the resources in the middle column by the recent deviant-evoked PS. Also, although somewhat larger than the PSs of the first type, PSs of the second type were still of relatively small amplitude and thus limited in their ability to propagate away from the column in which they were initiated (cf. Fig 3D, right, red and blue traces, and also Fig 3B within the green frame for the different ranges of propagation of PSs evoked in the standard and deviant columns).

SSA in the model was not related to preference of the middle column for one tone or another. This was verified by presenting the two tones with equal probabilities of occurrence, as well as switching their roles as standard and deviant (Fig 3F, top row, displays the average responses of these three conditions). Each tone evoked an average response depending mostly on its probability. When the two tones were presented with a probability of 50%, both evoked similar average response. These results reproduce the phenomenon seen in electrophysiological recordings, where responses depended on tone probability (Fig 3F, bottom row; data from Taaseh et al. [4]): Responses to a tone in the deviant condition were stronger than responses to the same tone in the equal condition, and those were in turn stronger than responses to the same tone when standard.

### Deviance sensitivity

**Fig 4:**
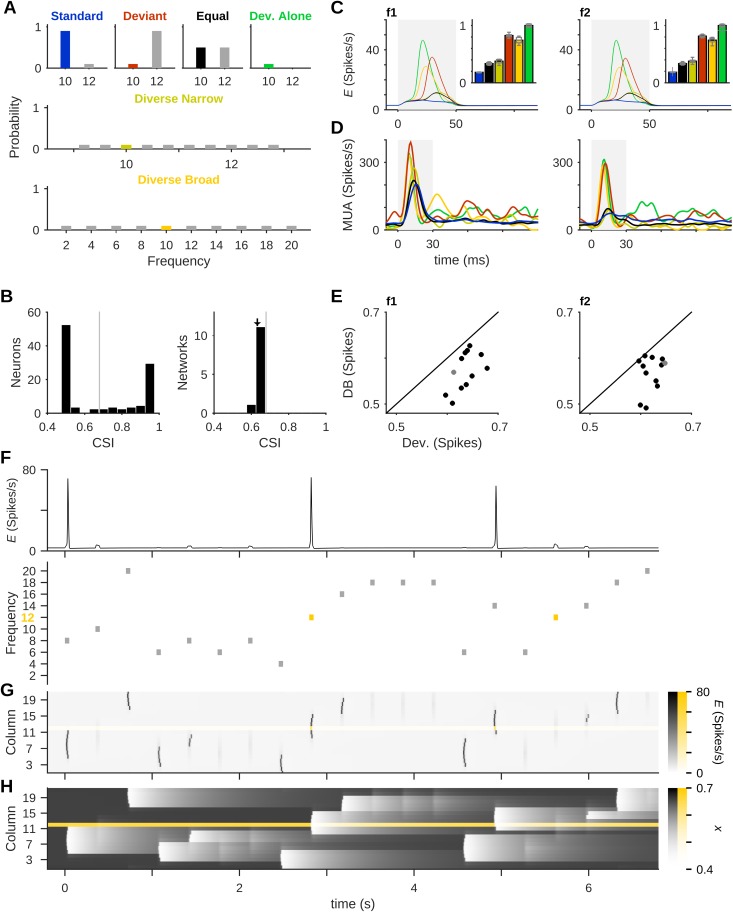
Sensitivity to deviance vs. rarity. Several control protocols were used to check whether the deviant response is due to its rarity alone, or alternatively to the violation of the regularity set by the standard. (A) There are 6 conditions in which a tone, e.g. f1 = 10, may be presented: As the Standard or Deviant in the regular oddball protocol; one out of two tones with equal probabilities (Equal); a deviant presented alone amid periods of silence (Deviant Alone); or a “deviant among many standards”, with multiple tones spanning an either narrow or broad range of frequencies (Diverse Narrow & Diverse Broad, respectively). Bar graphs show the probabilities of occurrence in the different protocols. (B) Left: A histogram of CSIs of single neurons in the middle column of an example network (gray line marks the mean CSI). Right: A histogram of CSIs calculated from the mean firing-rate in the middle column for 12 networks (see Eq 9). The mean single-neuron CSI of the network from the left-hand panel is marked with a gray line, and the CSI calculated from the average response of all neurons is marked by an arrow. (C) Average responses to f1 (left) and f2 (right) in the six conditions (peri-stimulus firing-rate, with stimulus duration marked in gray). Insets: Total spike counts, normalized to the Deviant Alone condition. Data are averages over 12 networks with different randomizations of tuning curves, each presented with 10 blocks of each protocol. (D) An example for multi-unit activity (MUA) responses from one site in rat auditory cortex to two frequencies (f1 = 13.3 kHz, f2 = 19.2 kHz) presented in the 6 conditions. Data replotted from Taaseh et al. [4] (the responses are from the same site used for Fig 3F, with the additional three conditions). (E) Spike counts for the Diverse Broad (DB) vs. Deviant responses of the 12 networks averaged in (C) to f1 (left) and f2 (right). The example network from (B) is marked with a gray dot. Responses to the Deviant condition were significantly stronger. (F) An example of the responses in the middle column (mean firing-rate, top) during a Diverse Broad protocol (bottom; occurrences of frequency 12 are marked in yellow). (G) and (H) Mean firing-rates (G) and synaptic resources (H) across multiple columns during the same protocol as in (F). Column 12, whose responses to presentations of its best frequency are highlighted in (F), is shown with a yellow colormap. Stimulus durations are marked in gray. In all simulation protocols, *A* = 5 spikes/s and ISI = 350 ms.

True deviance sensitivity requires more than just larger responses to rare stimuli: it is necessary that responses to deviant tones depend also on the identity of the other stimuli in the sequence. The extent of deviance sensitivity in our model was assessed using control protocols similar to those used in actual experiments [4,12]. These protocols were adapted from human studies [e.g. 44], and included two types of sequences (see Fig 4A and the Materials and Methods section):

1. The Deviant Alone protocol used only one tone, presented with the same probability as the deviant in the regular oddball protocol. All other trials consistent of continuous silence. This sequence provided an upper bound on the responses of the network to pure tones.
2. “Deviant-among-many-standards” sequences consisted of many equiprobable tones including the two tones tested in the oddball sequences. These sequences included the two main tones with the same probability as the deviant in the oddball sequence, but also many other tones that occurred each with the same probability. Thus, in these sequences there was no violation of expectations, in contrast with the oddball sequence in which the deviant violates the regularity set by repetition of the standard. In the Diverse Narrow protocol, tones spanned a frequency band similar in width to the one adapted by the regular oddball protocol. The Diverse Broad protocol, which is largely equivalent to the ‘control’ condition of Jacobsen and Schroger [44], is designed to control for simple sensory adaptation of the frequency band surrounding the standard and the deviant, while still avoiding any regularity of the tone sequence.

These three controls, together with the oddball and Equal protocols, gave rise to 6 conditions in which tones were tested (Fig 4A). Since our model includes some random heterogeneity in the tuning-curves within each column, we ran all the protocols on 12 networks with different randomizations of the tuning curves. The Common-contrast SSA Indexes (CSIs, defined in Eq 9) of the single neurons within the middle column of each network were all positive (Fig 4B, left). Their distribution was bimodal, related to the background input received by each neuron (see the Materials and Methods section for full description of the background inputs). Neurons that received strong, positive background input tended to respond strongly to standard tones and accordingly had relatively low CSI. Neurons that received negative background input and no sensory input had almost no standard responses but participated in the population spikes evoked by deviants, and accordingly had a high CSI. The CSI calculated from the mean firing-rate responses, averaged over all the neurons of the middle column, was slightly different from the mean single-neuron CSI (CSI = 0.632 based on the mean firing-rate vs. 0.679 ± 0.204 from single-neuron responses, marked by an arrow and a gray line in Fig 4B, right, respectively). Throughout the paper we calculate the CSI from the mean responses of all neurons in a column (rather than calculate CSIs of each neuron and then average). A histogram of the mean firing-rate CSI is shown in Fig 4B, right. Importantly, this histogram is very narrow (CSI = 0.643 ± 0.007, mean ± standard deviation). Our 12 networks therefore showed a similar level of SSA, despite the different randomizations of tuning-curves and the wide CSI distribution in the individual neurons of the middle column of each network.

Average responses of the middle column to tones in all 6 conditions are plotted in Fig 4C. An example of multi-unit activity (MUA) responses in rat auditory cortex to tones presented in these conditions is displayed in Fig 4D for comparison. These data were collected by Taaseh *et al*. [4], and are representative of the responses of both multiunit clusters and single neurons in rat A1, as shown by Hershenhoren et al. [12].

In the model, as in the experimental data, three conditions tended to give rise to relatively small responses: Standard, Equal and Diverse Narrow. In these conditions, the tones had high probability (Standard and Equal) or were packed in a narrow frequency band, causing substantial cross-frequency adaptation (Diverse Narrow). Three conditions evoked large responses: the Deviant Alone condition evoked the largest responses, as expected; and most importantly, responses to the Deviant condition were larger than those evoked by the same tone presented as part of the Diverse Broad sequence (cf. scatters in Fig 4E; paired *t*-test: Deviant > Diverse Broad, *t* = 6.4, *df* = 11, *p* = 5.3x10^-5^ for f1 and *t* = 4.9, *df* = 11, *p* = 5.1x10^-4^ for f2). Thus, the model passes the accepted test for true deviance sensitivity. Significantly, these simulation results are in line with the findings in rat auditory cortex by Taaseh et al. [4] (cf. Fig 4D) and Hershenhoren et al. [12], who showed this pattern of responses in about half of their neurons.

Why were the responses in the Deviant condition larger than in the Diverse Broad condition? This requires consideration of the responses in the deviant column in these two conditions. In the Deviant condition, the standard tone provided some weak input to the deviant column, causing some adaptation and reducing the average deviant response relative to the Deviant Alone condition. However, since most standard presentations did not evoke PSs in the deviant column, the adaptation caused in this column by standard tones remained small (cf. the response to deviant tones in Fig 3A relative to the response to the first standard tone, which should be similar to the Deviant Alone condition for f2). In contrast, in the Diverse Broad condition the probability of a tone to evoke a PS in its column was much higher, due to the wide distribution of tone frequencies in the sequence (Fig 4F–4H). These PSs propagated across the network according to its recent history, and some reached the deviant column and produced PSs in it. In consequence, PSs occurred in the deviant column at a rate that was higher than during an oddball protocol. This left the deviant column generally more adapted in the Diverse Broad condition than in the oddball protocol, and less able to generate responses that could propagate into the middle column. As an example, notice the PS generated in column 14, just before time *t* = 5 s in Fig 4G. This PS propagated into the deviant column and left it too adapted, such that it could not generate a PS in response to the next presentation of its own best frequency, f2 = 12, two stimuli later. Based on our results we conclude that the regularity of standard presentations in the oddball protocol effectively resulted in less adaptation in the deviant column compared to the Diverse Broad condition, and consequently the deviant responses in the middle column were stronger than Diverse Broad responses.

### The model reproduces the parameter dependence of experimental SSA

Our model reproduces the experimental dependences of SSA on several parameters: The probability of deviant occurrence, P; the frequency separation, Δf (defined here as Δf = f2 –f1); inter-stimulus interval (ISI); and input amplitude *A*.

**Fig 5:**
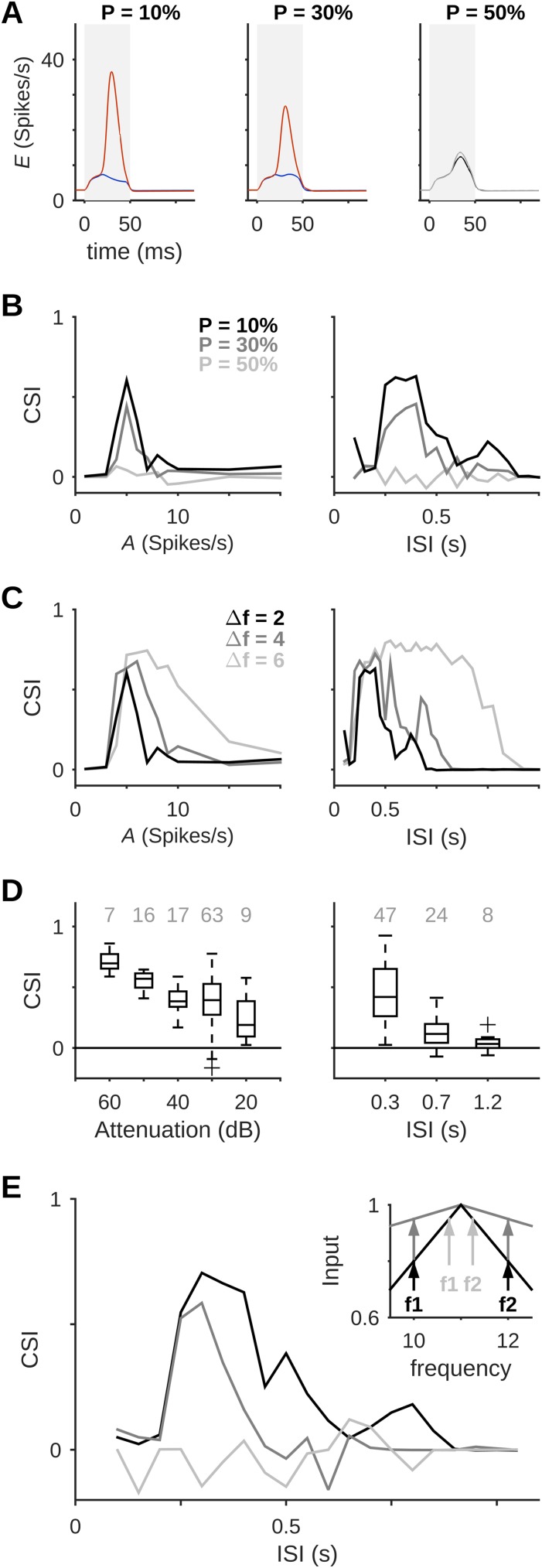
Properties of SSA. (A) The average response to the deviant tone increased as its probability of occurrence (P) was decreased. Stimulus durations are marked in gray. (B) SSA was present only within a certain range of input amplitudes (*A*, left) and inter-stimulus intervals (ISI, right). This range was slightly wider for smaller P. (C) Larger frequency separation (Δf) increased the CSI, and allowed SSA to occur over a wider range of input amplitudes (left) or ISIs (right). (D) Examples for the dependence of experimental CSI on stimulus parameters: Left, results of LFP recordings made by Taaseh [45] at different sound intensities (here represented as attenuations); right, intracellular recordings from Hershenhoren et al. [12]. Numbers above the box plots show the number of recording sites or cells for each attenuation or ISI, respectively. (E) Hyperacuity of SSA in the model is demonstrated using a network with tuning curves 10 times wider than Δf (*λ* = 20; see Methods). CSIs were lower and the range of ISIs was more limited (dark gray) compared to those of a network with normal tuning curves (*λ* = 5, black). Using normal tuning curves but smaller Δf gave no SSA (light gray). Inset: Scheme of tuning curves and stimuli for hyperacuity tests (colors as in the main plot). The *y*-axis shows input amplitude normalized to that of the best frequency. Default values for all simulation protocols shown here were P = 10%, Δf = 2, *A* = 5 spikes/s, ISI = 350 ms.

Smaller probabilities of deviant occurrence produced larger firing-rate responses to the deviant tone (Fig 5A). This resulted in increased CSI, as in Ulanovsky et al. [1]. In addition, SSA was present only within a limited range of input amplitudes (Fig 5B and 5C, left-hand panels), which was about 60% wider at the lower deviant probability (Fig 5B, left). As the firing-rates in thalamocortical fibers are related to sound intensity, the decreased SSA with increased input amplitude in the model may correspond to the experimental findings of Taaseh [45], who reported a decrease in CSI for increased sound level. Their findings, based on LFP recordings in rat auditory cortex, are re-plotted here in Fig 5D (left). A general decrease in CSI for increased sound level was also reported recently by Nieto-Diego and Malmierca [42], who recorded MUA responses across different fields of rat auditory cortex.

SSA was non-monotonic not only as a function of input amplitude, but also as a function of inter-stimulus interval (Fig 5B and 5C, right-hand panels). Here, the range of inter-stimulus intervals allowing SSA was about 16% wider at lower deviant probabilities (Fig 5B, right). This is also in line with experimental results [1,4,12]; an example for the experimental dependence of CSI on ISI is provided in Fig 5D (right), which re-plots the results of intracellular recordings by Hershenhoren et al. [12].

Increasing the frequency separation between standard and deviant, Δf, usually produced an increase in CSI, extending the ranges of input amplitudes and inter-stimulus intervals that allowed SSA (Fig 5C). In general, SSA was present only for rather short ISIs (< 1 s), except for the case of Δf = 6 that showed SSA for ISIs up to nearly 2 s, similar to the results of Ulanovsky et al. [1] at their largest Δf, as well as Hershenhoren et al. [12]. The effect of increasing the frequency separation on CSI in columns other than the middle column is presented below, as part of the experimental predictions of the model.

Our model showed hyperacuity, which is another important feature of SSA in A1 [1]: SSA was present for Δf values substantially smaller than the width of the tuning curve. We first tested for hyperacuity by choosing f1 and f2 such that Δf was 10 times smaller than the input tuning-curve width. This setting put the two tones near the peak response frequency of the middle column, so that this column was adapted by both the standard and the deviant. Consequently, it couldn’t support the initiation of population spikes and did not show any SSA (Fig 5E, light gray trace). This result emphasizes the fact that SSA in our model depends on the propagation of PSs between columns, rather than depression of thalamocortical synapses in the middle columns. As an alternative method for testing hyperacuity, we increased the frequency resolution of the model. This was achieved by increasing the width of the input tuning-curves. For these simulations, the width of the input tuning curve was set to 20 times the frequency difference between two nearby columns. In consequence, the distance between the two columns used for standard and deviant, Δf = 2, corresponded to 1/10 of the thalamocortical tuning-curve width. Such a setting corresponds to finer columnar organization, i.e. shorter-distance connections within the cortex. SSA was present in this paradigm, albeit with lower CSI values and in a narrower range of ISIs compared to the standard network (Fig 5E, dark gray vs. black traces).

### SSA is due to an interplay between different response patterns to standards and deviants

**Fig 6:**
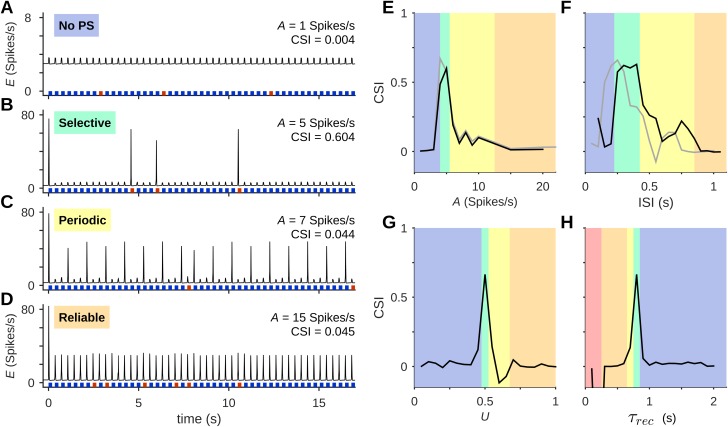
Regimes of response limit SSA to a narrow range of parameters. (A-D) Responses to oddball sequences always fell into one of 4 general patterns (regimes). These are demonstrated here with different input amplitudes: (A) No PS to either standard or deviant; (B) Selective PS response, where PSs were evoked mostly by deviants; (C) Periodic PS responses, where some standards evoked PSs and may have prevented PS responses to subsequent deviants; (D) Reliable PS response, with both standard and deviant evoking PS responses. CSI values are large only in the Selective regime. Colored bars under the traces represent presentations of standard (blue) and deviant (red) stimuli (bar widths are not to scale). (E-H) The same set of response regimes is observed when changing different stimulus or network parameters. The parameters are input amplitude *A* (E), ISI (F), and synaptic properties such as the fraction of resource utilization *U* (G) and the recovery time-constant *τ*_rec_ (H). Colors represent regimes as in (A-D). Gray curves in (E) and (F) show the parameter dependence in a network with homogeneous columns (all input-receiving neurons in each column have the same best frequency). In (H), the red area corresponds to spontaneous PS generation (“Bursting”). In all protocols, P = 10% and Δf = 2; default values were *A* = 5 spikes/s, ISI = 350 ms, *U* = 0.5, *τ*_rec_ = 800 ms.

SSA is a non-monotonic function of most parameters of the simulation (cf. Fig 5B and 5C). This property is due to the patterns of PSs evoked by the two tones in the oddball protocol. We identified four different regimes of PS initiation: (i) No stimulus was able to evoke a PS. This “No PS” regime occurred for low *A* values, when no stimulus was strong enough to elicit a PS (Fig 6A), as well as for short ISIs when synaptic resources were too depleted for PS initiation and propagation. It was also found at small values of *U*, the fraction of resources used upon synaptic activation, which effectively reduced synaptic strength, and for long recovery time-constants of the synaptic resources, which kept the synaptic weights dynamically small. This regime showed no SSA, and was named “No PS” since it was the only regime that had no PSs, except a single PS that sometimes occurred at the beginning of a protocol. (ii) Deviant stimuli evoked PSs in the middle column whereas standards did not. This “Selective” regime (Fig 6B) is the one that showed strong SSA, as illustrated in Fig 3. (iii) A Periodic regime, in which the standard tone evoked PSs in its column, and these invaded the middle column only once every few stimuli (Fig 6C). PS responses to the deviant were not always successful because the middle column, and sometimes the deviant column as well, were adapted by the PSs initiated in the standard column. (iv) Each and every stimulus evoked a PS, regardless of its identity (Fig 6D). This “Reliable” regime occurred for example at high input amplitudes and long ISIs. Regimes (ii) and (iii) are similar to the phenomenon of cycle skipping in excitable systems, where periodic excitation gives rise to responses in some but not all cycles.

Fig 6E–6H illustrate the association of strong SSA with the Selective regime. CSI is plotted against selected parameters, as in Fig 5, and the different response patterns, identified from the simulation traces, are superimposed in color. In addition to the stimulus parameters, *A* and ISI (Fig 6E and 6F, black curves), the same regimes and the same dependence of CSI on parameters occurred when varying dynamical parameters of the local intracortical connections such as *U*, the fraction of synapse utilization, and *τ*_rec_, the recovery time-constant of synaptic resources. Very short *τ*_rec_ gave rise to yet another pattern of activity, in which PSs were generated spontaneously. CSI was generally high only within the Selective regime, although there was also moderate SSA on the margins of the Periodic regime.

As mentioned above, one of the modifications we made to the model of [28] was to introduce heterogeneity in the tuning curves within each column, following experimental findings in mouse A1 [46,47]. We use the sensitivity of SSA to stimulus parameters to assess the effect of this feature of the network (Fig 6E and 6F). The same parameters were tested for a heterogeneous network, as was used throughout this work (black) and for one with homogeneous columns, where all the input-receiving neurons in each column have the same best frequency (gray; the regimes marked in color on the plot area are for the heterogeneous network). In the homogeneous network, SSA existed in a similar range of amplitudes and in a range of ISIs that included shorter values compared to the heterogeneous network. Thus, the basic mechanism of SSA was unaffected by the heterogeneity, which primarily contributed to the existence of SSA at longer ISIs.

**Fig 7:**
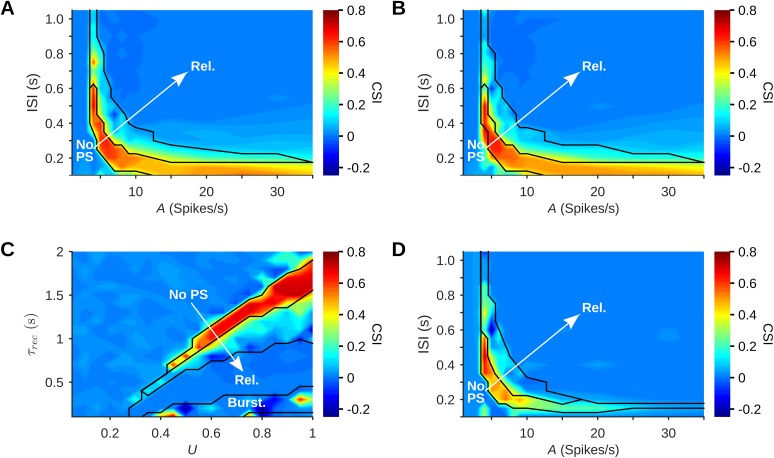
SSA is limited to a narrow region in parameter space. (A) CSI as a function of amplitude (*A*) and ISI (using *U* = 0.5, *τ*_rec_ = 800 ms). Here and in the other panels, an arrow marks the transition from the No-PS regime to the Selective, Periodic and finally the Reliable regime (Rel.), delineated by the black lines. (B) CSI in a network with the same parameters as in (A), but with homogeneous columns (all input-receiving neurons in each column have the same best frequency). Heterogeneity of the tuning curves acted to shift and slightly expand the range of parameters that showed SSA, and specifically its low-*A* branch. (C) CSI as a function of synaptic parameters *U* and *τ*_rec_ (*A* = 5 spikes/s, ISI = 350 ms). On this plane there was also a Bursting regime (Burst.), composed of networks that generate spontaneous PSs, crossing the Reliable regime. (D) CSI in a network with the same parameters as in (A), but without thalamocortical depression. Thalamocortical depression thus extended the range of parameters that showed SSA. In all protocols, P = 10% and Δf = 2. Default values were *A* = 5 spikes/s, ISI = 350 ms, *U* = 0.5, *τ*_rec_ = 800 ms.

It seems therefore that SSA exists in this model only within a relatively narrow range of parameters. To verify this claim generally, we searched exhaustively through the space of possible parameters of the stimulus sequence (amplitude and ISI) and through the space of possible network parameters (*U* and *τ*_rec_). Fig 7A presents the CSI for different combinations of *A* and ISI, with the different response regimes delineated on the map. Strong SSA is mostly confined to the Selective regime. The existence region of SSA has the shape of a narrow, curved band on the *A*-ISI plane. It consists of two “branches”, one spanning a wide range of ISIs but confined to low input amplitudes, and the other confined to short ISIs and spanning a wide range of input amplitudes. Fig 7B shows the CSI map and the regimes obtained in a homogeneous network, where all inputs to a column share the same best frequency. Comparison with Fig 7A reveals that network heterogeneity resulted in a slightly expanded low-amplitude, long ISI branch of the existence region for SSA.

We studied the existence region for other parameters as well. For example, when keeping the stimulus parameters fixed (*A* = 5 Spikes/s, ISI = 350 ms) and varying the synaptic parameters *U* and *τ*_rec_ (Fig 7C), SSA was also confined to a narrow band of parameters (Fig 7C). These calculations extend the results of Fig 6G and 6H. Here, the existence region for SSA was somewhat wider when both *U* and *τ*_rec_ were large.

### Subtractive and divisive processes in SSA

**Fig 8:**
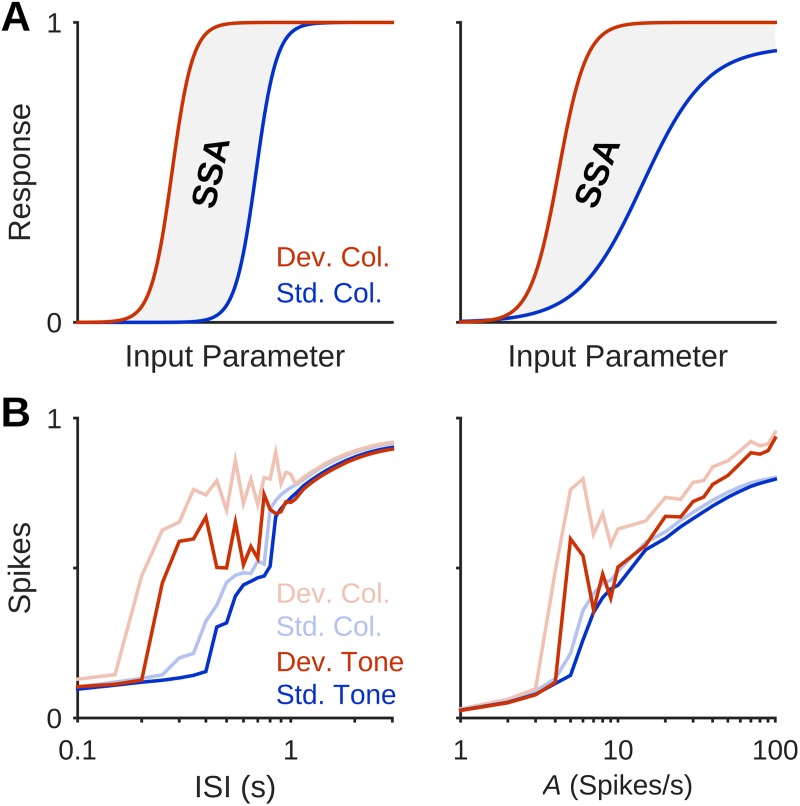
Adaptation shapes the SSA Region by subtractive or divisive operation on the input of column response curves. (A) Adaptation might affect the response curve of a cortical column in a subtractive (left) or divisive (right) manner. The potential SSA region lies between the response curves of the weakly-adapted deviant column and the strongly-adapted standard column. (B) Response curves of the deviant column and standard column, each to its own best-frequency stimulus (light red and light blue, respectively) for ISI (left) and input amplitude *A* (right). Red and blue curves show the responses within the middle column to the deviant and standard tones, respectively. Curves demonstrate the effect of adaptation, suggesting a subtractive operation on the logarithmic ISI-scale and divisive operation on the logarithmic *A*-scale. Responses to the deviant tone were attenuated more strongly in the process of propagating to the middle column compared to standard responses. In all protocols shown here, P = 10%, Δf = 2, *U* = 0.5, *τ*_rec_ = 800 ms.

The existence region found for SSA in our simulations can be viewed as a consequence of the difference between responses of the deviant and standard columns, which are affected differentially by adaptation. SSA exists when the slightly-adapted deviant column generates PSs while the strongly-adapted standard column doesn’t. Consider the responses of the deviant and standard columns as a function of any of the parameters of the model. Such a relationship is called here the ‘response function’. Adaptation modifies the response function, and its net effect can be heuristically modeled by a modification of the relevant model parameter. For example, adaptation as a function of sound level can be described by effectively reducing the sound level of the input. Operations on the input of the response function fall into two major classes [48], illustrated in Fig 8A: (i) Subtraction, which shifts the response function along the parameter axis while preserving its slope (left); and (ii) division, which scales the slope of the response curve (right).

In Fig 8B, the responses of the middle column, which is where the SSA was evaluated, to standard and deviant stimuli (blue and red) are displayed as a function of log(ISI) (left) and log(*A*) (right). These responses are largely inherited from the standard and deviant columns (where the PSs are generated; light blue and light red), although responses to deviants were attenuated more strongly in the middle column (red vs. light red) compared to the responses to standards (blue vs. light blue). The responses to standards showed a sigmoidal, monotonic dependence on both parameters. The response curves to the deviants were shifted to smaller values, and also somewhat distorted: for both parameters, there was a non-monotonic region corresponding to mid-range standard responses. Comparing these results of the simulation with the schematic curves in Fig 8A, we see that adaptation acts as a subtractive operation along the logarithmic ISI scale, and at least within the low-*A* monotonic region of the deviant responses, it is a divisive operation along the log(*A*) scale (Fig 8B).

While the divisive operation on stimulus amplitude doesn’t have an obvious explanation, the subtractive effect along the log(ISI) scale is easily explained by considering the effective ISI. On average, this was ISI/(1 – P) in the standard column, where P is the probability of occurrence of the deviant tone, but ISI/P in the deviant column. On the logarithmic scale, these become shifts of –log(1 – P) and –log(P) with respect to a column stimulated repetitively (P = 1). Interestingly, these shifts are the expressions for the objective surprise associated with the standard and deviant tone, respectively. Thus, at least under these conditions, the model roughly implements a predictive coder [49].

### The role of thalamocortical synaptic depression within the SSA region

Since the existence region for SSA is rather narrow, we were interested in mechanisms that may increase its extent. We show here that the depression of the thalamocortical synapse is one such mechanism, whose inclusion in the model increases the range of parameters producing large CSI (Fig 7D).

**Fig 9:**
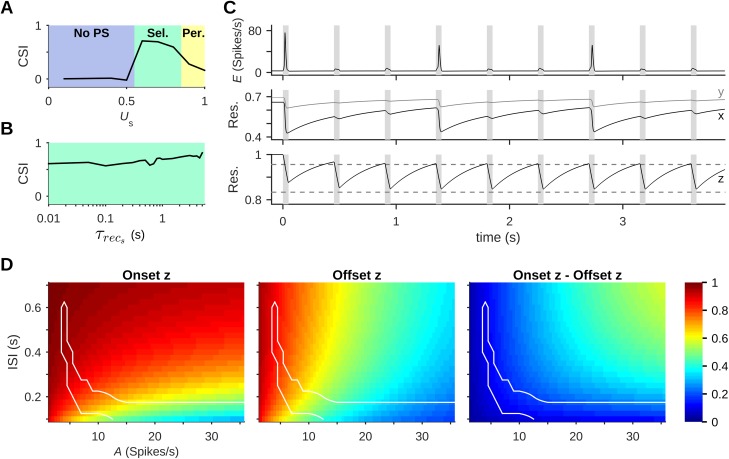
Thalamocortical synaptic depression. (A) Changing *U*_s_, the fraction of thalamocortical (ThC) resource utilization, showed three of the four response regimes shown in Fig 6, with SSA present mainly in the Selective regime. (B) Changing *τ*^s^_rec_, the time-constant of recovery in ThC synapses, did not take the network out of the Selective regime. (C) An example of periodic PSs in the standard column. Periodic PS responses (top) arise from recurrent network dynamics, as manifest in the dynamics of *x* and *y*, the mean fractions of available resources (Res.) in excitatory and inhibitory synapses, respectively (middle), and not from dynamics of the ThC synapses mediating the standard tone (*z*, mean fraction of available resources; bottom. Dashed lines show analytical values as in D). ThC dynamics show steady oscillations that are substantially faster than the period of PS responses. Stimuli presentations are marked in gray. (D) Onset (left) and offset (middle) *z* for the steady oscillations reached in the standard column (Eqs 17 and 18), computed analytically and plotted across the *A*-ISI plane. The difference between the two (right) is the fraction of ThC resources recovered during each ISI. Borders of the Selective regime are marked in white. In all protocols, P = 10%, Δf = 2, *U* = 0.5, *τ*_rec_ = 800 ms; default values were *A* = 5 spikes/s, ISI = 350 ms, *U*_s_ = 0.7, *τ*^s^_rec_ = 300 ms.

Changing *U*_s_, the fraction of resource utilization in the ThC synapses, produced a pattern similar to changing *A* or *U*: Low values gave rise to the No-PS regime and higher values to the Selective and Periodic regimes (Fig 9A). In contrast, changing the value of *τ*^s^_rec_, the time-constant of recovery of the ThC synapses, did not abolish the Selective PS responses (Fig 9B). The only effect of increasing *τ*^s^_rec_ was a slight increase in CSI. Robustness to changes in *τ*^s^_rec_ is highlighted by the fact that tested values spanned over two orders of magnitude.

The dynamics of ThC depression in our model (Eq 5 in the Materials and Methods section) can be treated analytically. When presenting a sequence of identical, repetitive stimuli, the mean fraction of available resources in the ThC synapses (denoted by *z*) is periodic with a period that equals the inter-stimulus interval (Fig 9C, bottom). This is approximately what happens during a long sequence of standard stimuli. Importantly, treating the system as an iterated map reveals that *z* assumes the same value at the onset of each stimulus (onset *z* value) and another value at the offset of each stimulus (offset *z* value). These values can be derived analytically under some approximations (Eqs 17 and 18; resulting values shown as dashed lines in Fig 9C, bottom). Importantly, the analytical derivation further reveals that this is the only steady-state behavior of the map, so that the resources of the ThC synapses cannot show oscillations with longer periods.

Thus, ThC depression cannot account for the more interesting dynamical phenomena in the network. For example, in Fig 9C the population spikes within the standard column occur once in every three stimulus cycles, while the dynamical parameters of the ThC depression only follow the faster periodicity of a single stimulus cycle. Similarly, ThC depression alone cannot produce the phenomenon we observe in the Periodic regime, in which PSs may occur in the middle column on some but not all stimulus presentations (Fig 6C). In both cases, Periodic PS responses to trains of standard stimuli resulted from the dynamics of the synaptic resources of the intracortical connections (denoted respectively by *x* and *y*). Indeed, while *z* follows the rate of stimulus presentation, *x* and *y* generally follow the slower rate of the population spikes.

We use the onset and offset *z* calculated analytically (Eqs 17 and 18) to examine the effects of ThC depression within the SSA Region. The SSA Region found in our simulations consists of two “branches”, in which ThC depression plays different roles (Fig 9D).

The first branch, at low stimulus amplitude and spanning a wide range of ISIs, has high values of both offset and onset *z*. The equations show that the low-level stimuli that gave rise to this branch in the simulations hardly deplete ThC resources, and the moderate ISIs allow recovery of virtually all the depleted resources. Thus, ThC depression has little effect on network responses in this branch, explaining the robustness of SSA with respect to *τ*^s^_rec_ found in our simulations (Fig 9A).

The second branch lies in the short-ISI range and spans a wide range of stimulus amplitudes. In this branch, the equations show that stimuli cause significant ThC depletion (low offset *z*) and little resource recovery (low onset *z*). This combination causes a strong effective attenuation of stimulus amplitude, explaining why in the simulations the CSI values were quite uniform (Fig 7A)–high-amplitude stimulation was effectively attenuated to input strength that was comparable to that of low-amplitude stimulation. The analytical treatment suggests therefore that this branch is formed by “stretching” the short-ISI, medium-amplitude region into the high-amplitude range. We note that both branches of the SSA Region have a similar, rather negligible amount of resources recovered during the ISI (Fig 9D, right-hand plot).

To summarize, the main effect of thalamocortical depression in our model is to weaken the dependence of CSI on some model parameters. This is illustrated in Fig 9 for input amplitude–at least at short enough ISIs, ThC depression adaptively reduces stimulus amplitude and therefore brings the activity pattern into the selective regime. The same analysis is also true for the effect of the fraction of resource utilization in the thalamocortical synapses (*U*_s_) on SSA, which was found to resemble that of stimulus amplitude (cf. Figs 6E and 9A): At the low-level, long-ISI region *U*_s_ indeed strongly affected SSA. However, within the short-ISI branch, SSA should be only weakly dependent on *U*_s_, which effectively acts as a modifier of stimulus amplitude.

Thus, ThC depression has an important role in shaping the SSA Region itself, expanding the parameter region formed by intracortical depression alone. This finding was confirmed by our simulations of the same network as the one used for Fig 7A, except that ThC depression was removed. We tested this network on the same range of stimulus parameters (Fig 7D): The Selective regime without ThC depression was narrower than that of the standard network, and while it showed strong SSA in the low-*A* branch, SSA was weak and existed for a more limited set of parameters on the short-ISI branch.

### Further experimental tests of the model

**Fig 10:**
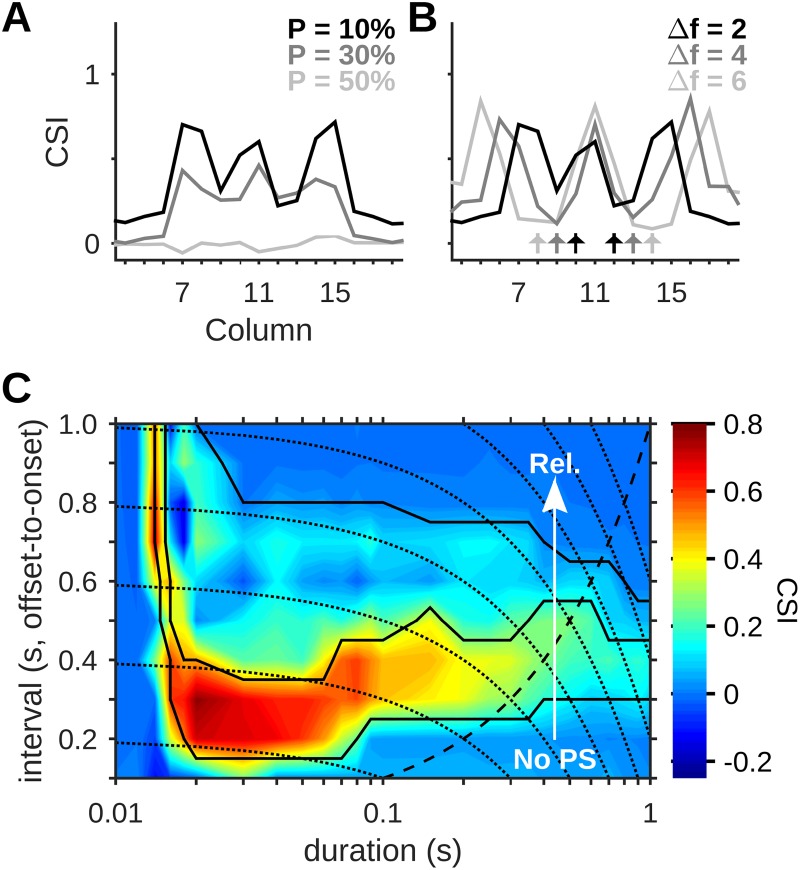
Predictions for experimental studies. (A,B) SSA was strong in the middle column (no. 11) and considerably weaker in the columns most sensitive to the stimulus frequencies. In columns farther away from the middle, the CSI was high but dominated by one of the two conditions (e.g. low-index columns showed high CSI mainly due to strong responses to f1 as the deviant). Dependence of CSI on column is shown for different values of P (A) and Δf (B). Arrows in (B) mark the different pairs of frequencies presented. (C) Color map of CSI values for different combinations of stimulus duration and offset-to-onset interval. Solid lines show the borders of the different response regimes. An arrow shows the transition from the No-PS regime to the Selective, Periodic and finally the Reliable regime (Rel.). Dotted lines mark constant ISIs (onset-to-onset). The dashed curve marks the line on which the interval and duration are equal. Default values were P = 10%, Δf = 2, *A* = 5 spikes/s, ISI = 350 ms.

Our simulations of a network with multiple columns allowed us to study model responses to the oddball protocol in columns other than the middle column. For these columns, the two tones presented were usually located on the same side of the peak of the tuning curve (Fig 10A and 10B).

We found that in columns whose best frequency corresponded exactly to either the standard or the deviant, SSA was considerably weaker than in the middle column. This is because adaptation to the best frequency as the standard prevented PS responses to deviants from entering the column, while responses to the best frequency when it served as deviant were attenuated by cross-frequency adaptation. It should be noted that when Δf is smaller than 2, the smallest value shown in Fig 10B, the middle column shows weak SSA as well (cf. Fig 5E).

In contrast, in columns with a best frequency either lower than f1 or higher than f2, SSA could be even stronger than in the middle column. This resulted from large deviant responses in one of the protocols. For example, columns whose best frequency was lower than the low frequency of the oddball sequence (f1) showed large responses to f1 as deviant but not to f2 as deviant. Responses to f2 as deviant in these columns were small because PSs evoked in the f2 column had to travel through the strongly adapted f1 column before reaching the low-best frequency columns. The CSI in some of the low-best frequency columns was even higher than in the middle column because deviant responses to f1 suffered less cross-frequency adaptation from the standard, which was farther away on the frequency scale. The opposite occurred on the other side of the network.

The results described here correspond to playing an oddball protocol with f1 and f2 not centered on the best frequency of the recording site. Such a paradigm has not been systematically studied in cortical recordings.

The model makes clear predictions for the effect of tone duration on SSA. Fig 10C presents a map of CSI values as a function of tone duration and the offset-to-onset interval. The latter quantity is highly relevant for the dynamical behavior of the network, as it is the time allowed for recovery of synaptic resources. High CSI values in Fig 10C are limited to shorter tone durations and offset-to-onset intervals, and SSA was always abolished at long tone durations. However, the reason that SSA disappeared at long tone durations depended on the offset-to-onset interval: At long offset-to-onset intervals, longer tone durations produced more frequent PS responses (Periodic and Reliable regimes). This was due to the ongoing recovery of intracortical resources, showing that thalamocortical depression did not play an important role at these intervals (cf. the high onset *z* for long ISIs in Fig 9D). In contrast, at short offset-to-onset intervals, SSA was abolished at long durations because no PSs were elicited. We attribute this to the accumulating depletion of thalamocortical resources from one stimulus to the next, which rendered them too low for eliciting PS responses even in the deviant column.

We note that at intervals around 300 ms, used in most of the simulations above, SSA was quite robust to changes in duration but not to changes in the interval. Indeed, Hershenhoren et al. [12] recently reported that in rats, while CSI is significant for tone duration of 30 ms and offset-to-onset intervals of 270 ms, it becomes rather weak at intervals of 670 ms and 1170 ms (cf. Fig 5D, right).

## Discussion

We showed here that a network with recurrent dynamics and synaptic depression is capable of producing stimulus-specific adaptation. In particular, the network demonstrates true deviance sensitivity, in contrast with competing models based on feedforward synaptic depression [4,25] but in accordance with experimental results [4,12].

### Population spikes in auditory cortex

SSA in our model is first and foremost a network phenomenon that does not require thalamocortical depression. It depends on intrinsic cortical dynamics that produce population spikes and allow their lateral propagation across the network. The existence of population spikes in A1 has been inferred from the distribution of EPSP amplitudes [29,30], multi-electrode recordings [31–33], and network [34] and single-cell calcium transients [35]. *In-vitro* recordings also show population events occurring in A1 upon stimulation of the thalamus [50]. The evidence suggests that large ensembles of neurons may be active during such events: Bathelier et al. [35], for example, used 2-photon calcium imaging with single-cell resolution and found that the population events dominating the activity in A1 included a large fraction (over half) of the imaged neurons.

The PSs in our model are of slightly faster rise-time and shorter duration relative to evoked population bursts recorded in rat A1 [31–33]. While the underlying mechanisms have not been identified, modeling work [36,37] has suggested that population spikes may be a consequence of the dynamics of a network with depressing synapses. Loebel et al. [28] further demonstrated that such networks can be used to provide a unified account for many response properties of neurons in auditory cortex, including forward masking, lateral inhibition and hypersensitive locking suppression. Here we showed that such a network can produce SSA as well.

### A simplified network model of A1

The model we presented here, similar to [28], should be taken as a simplified representation of A1. The use of a rate-model to represent single neurons in A1 was introduced by Loebel et al. [28], based on a previous work that showed the equivalence of such a model to a firing-rate description of a recurrent network with synaptic depression in terms of its activity [36]. The model is stripped of many details such as layers, feedforward inhibition, cell types and numbers, accurate connectivity probabilities, or realistic connectivity profiles. The simplifications made it possible to focus on a minimal number of basic features and demonstrate that they are sufficient to support cortical SSA. Nevertheless, the model keeps some crucial features of the actual rodent auditory cortex.

In cortex, connection strengths and probabilities decay smoothly with spatial distance [27]. In the model, this organization was discretized into columns. In rat A1, the tonotopic gradient spans about 2 mm and covers about 7 octaves [40]; in mouse A1 the tonotopic axis is somewhat shorter. Since the model had 21 columns, each corresponded to about 1/3-1/2 octave. Tuning curves in the model have a half-maximum width of 5 columns, or 1–2 octaves. This is similar to curves measured in cortical recordings [40]. Based on the above numbers, the usual frequency separation of our simulations (Δf = 2) corresponds to about 2/3 of an octave, or slightly above the typical frequency separation used in experiments, which is 0.53 octave (the 44% of [4,12] and 0.37 of [1]). Thus, the main conditions of the simulations reflected the typical experimental conditions used to study SSA.

The connection strengths within each column were set to prevent spontaneous PS activity (see Materials and Methods). While it is difficult to compare between synaptic strengths in a rate-model and electrophysiologically-measured quantities, our tests of single-neuron activation in the model showed that connections between excitatory neurons within the same column were weaker than those reported *in-vitro* in mouse A1. On the other hand, connections were more probable [27], since our network had full connectivity within each column (see Materials and Methods). Synaptic depression parameters used here are on the same order as those reported in A1 and used in modeling its activity (*U* = 0.55, *τ*_rec_ = 500 ms in [51], *U*_s_ = 0.8, *τ*^s^_rec_ = 1 s in [52]).

Finally, following [28], our model features purely feedback inhibition, where inhibitory neurons do not receive sensory input. Feedforward inhibition is known to have an important role in A1, arriving at a typical delay of a few milliseconds following the excitatory (presumably thalamocortical) currents [38], presumably due to the additional synapse on the way. Adding an explicit delay would significantly complicate our rate model (see e.g. [53] for an implementation of such a delay). We chose not to implement a synaptic delay, instead emulating the effect of delayed inhibition by feedback inhibition. Indeed, in simulations in which inhibitory neurons also received sensory input, the resulting responses were similar to those of the main simulations as long as the direct sensory input to inhibitory neurons was relatively weak. With stronger inputs to the inhibitory neurons, responses were abolished due to the simultaneous arrival of excitatory and inhibitory inputs onto the neurons in the network.

The absence of direct feedforward inhibition should not affect our conclusions regarding the role of synaptic depression in shaping SSA, based on the following reasons: (i) The first evoked spikes in the thalamo-recipient neurons are already sufficient to trigger the population spike, with feedforward inhbition having its effect only following these initial spikes [38]. Furthermore, once a population spike is triggered, its propagation involves feedforward inhibition, since the intercolumnar connections target both excitatory and inhibitory neurons. Therefore, feedforward inhibition should not prevent the initiation of population spikes. (ii) Synaptic depression would come into play once the first spike is fired, and is the main factor that prevents spiking over the longer time-scale, i.e. for inter-stimulus intervals longer than 100 ms [54]. (iii) The feedforward inhibition itself undergoes SSA, thus scaling with the excitatory responses and at most modulating the SSA, not generating it [55]. Some insight into the effects of feedforward inhibition on SSA may perhaps be gained from the model of Schiff and Reyes [52]. Their results suggest that at the low stimulation rates used in the oddball protocol, feedforward inhibition may balance the effects of thalamocortical depression and result in a relatively constant net drive to the excitatory neurons. Such an effect would emphasize the role of depression of the recurrent synapses in generating SSA (cf. Fig 7D, which shows the extent of the SSA region without thalamocortical depression).

### Sensitivity to stimulus and network parameters

The main weakness of our model is its sensitivity to stimulus and network parameters (Figs 6, 7 and 9A). The existence range of SSA in parameter space is expanded by mechanisms such as depression of the thalamocortical synapses and, to a lesser extent, heterogeneity of the tuning curves. However, even with the action of the above mechanisms, SSA in our model is not a robust phenomenon. The sensitivity of SSA to network and stimulation parameters stems from its existence in the intersection region of the requirements of PS response to the deviant tone but no PS response to the standard tone (as illustrated by the response curves in Fig 9). The extent of this region is determined by the constraints posed by the frequency-tuning of columns and by the lateral propagation: Propagation should be strong enough to deliver deviant responses into the middle column, yet weak enough to prevent most of the successful standard responses from doing so.

For the model to be a viable account of cortical SSA it is necessary to assume the existence of tight regulation of the cortical network, which drives it into the SSA Region. One possible locus of such regulation may be the narrow dynamic range of firing-rates in thalamocortical fibers [9] and/or thalamocortical EPSPs in layer-4 input neurons [56], which in our model would both correspond to a limited range of input amplitudes. Sources of regulation might include neuromodulatory control, a mechanism that could conceivably drive A1 between states favoring SSA and states favoring other computational tasks. Cholinergic input, for example, may control important parameters such as the utilization parameters for sensory and recurrent synapses [57–59].

### Comparison with other models

Two other classes of models for SSA have been studied previously. The first class of models is based on depression of the feedforward synapses [4,12,25,26]. Thalamocortical depression was not essential for the existence of SSA in our model, so that the mechanism studied here is indeed different from the SSA that depends on feedforward depression. The second class of models consists of networks with recurrent connections. The emergence of SSA from synaptic depression in the local, cortical connections has also been shown in a recent computational work by May et al. [20]. Their model is different from ours in that they ignored the single neurons, modeling the interaction between cortical columns. Their analysis is not detailed enough to understand whether the basic mechanisms that produce SSA in their model are the same as in the model presented here. Furthermore, they provide only limited information on the dependence of SSA on stimulus and network parameters. A different network model of SSA-like responses has been developed by Wacongne et al. [21]. Their model depends on synaptic dynamics, although not on synaptic depression. However, their model has been developed in order to test a specific model of predictive coding, and some parts of it (e.g. the short-term memory component) are hand-crafted to have the required properties. Thus, it is different from the network model we describe here, which uses generic network mechanisms to produce SSA. In consequence, all further comparison with existing models considers only feedforward models of SSA.

### Comparison with experiments

Our model correctly accounts for a number of properties of SSA in rat auditory cortex. SSA increases with frequency separation between the tones and with decreasing probability of the deviant [4,12]. SSA decreases at high sound levels [45]. Similarly, SSA weakens as ISI is increased [4,12]. The model suggests that SSA should also decrease at low amplitudes and very short ISIs. The low-amplitude and short-ISI ranges are presumably overlooked in experimental studies because of the difficulty to elicit responses with such parameters. All of these properties are also correctly predicted by other models of SSA [25,26]. On the other hand, in the model presented here, single-trial responses to standard tones show occasional successes, consistent with a recent intracellular study of SSA [12], where the largest responses to the standard tone were as large as those in the Deviant and Deviant Alone conditions. The existence of occasional strong responses to the standard tone may also find support in the spike-count distributions in [43], where successful standard responses were similar in strength to the deviant responses. Feedforward models cannot easily account for such findings.

Importantly, the model has true deviance sensitivity, in which responses to deviants within a regular background were stronger than to deviants within an irregular background (“many standards”, Jacobsen and Schroger [44]). This phenomenon could not be explained by a feedforward model of adaptation in narrow frequency channels [4], at least not when frequencies were close enough to produce cross-frequency adaptation. Mill et al. [25] also showed a small preference for true deviants over the “deviant-among-many-standards”, but only at large frequency separations (equivalent to separations larger than our Δf = 2). At these frequency separations, the deviant and standard were far enough apart that the standard in the oddball sequence did not induce strong cross-frequency adaptation of the deviant responses, while the many-standards condition did [25]. In contrast, in our model the many-standards (our Diverse Broad) condition generically gives rise to cross-frequency adaptation that is stronger than in the oddball sequence. The many-standards sequence, with its extensive propagation of population spikes across the network, caused more activity in the deviant column than the oddball sequence, and accordingly also more adaptation. Consequently, the deviant column was able to produce stronger responses to its best-frequency tones during the oddball sequence than during the many-standards sequence. Neuronal responses in rat auditory cortex indeed often show true deviance sensitivity [12].

### Experimental predictions

In addition to modeling existing data, the model makes predictions that have not yet been tested in cortex. In our model, the CSI depends on the positions of the two tones relative to the column’s best frequency, with a minimum CSI when one of the tones is equal to the best frequency. To our best knowledge, this prediction has not been tested systematically in cortical recordings. Interestingly, such conditions have been tested in the inferior colliculus by Duque et al. [60]. Their results are qualitatively different from our prediction for cortical SSA in that they do not show a decreased CSI for f1 or f2 equal to the best frequency; rather, they are consistent with purely feedforward depression and the characteristic asymmetry of tuning curves in the auditory system. Bäuerle et al. [11] have also looked at SSA with frequency pairs not centered on the site’s best frequency, in gerbil ventral MGB. Most of these results favor the feedforward depression mechanism. Thus, in subcortical stations, feedforward depression may be the dominant mechanism shaping SSA.

Our model predicts that SSA should be found within a limited range of stimulus durations (cf. Fig 10C, the longer-duration “branch”). Significantly, our prediction contradicts that of a model based on feedforward depression [26], which suggests that SSA should become stronger when stimulus duration is extended. We know of no study that checked systematically the dependence of SSA on stimulus duration. However, there is a tendency to use relatively short stimuli in SSA experiments in rats (30 ms, [3,4,12]; 75 ms [5,9,60]), suggesting that indeed longer stimuli result in less SSA.

### Applicability to other modalities

SSA has been demonstrated in somatosensory and in visual cortices. Mechanisms similar to those studied here may be at play in the generation of SSA in barrel cortex. In rat barrel cortex, SSA has been reported for whisker identity as well as for velocity and direction of whisker deflection [61]. The responses to non-principal whiskers are probably mediated by lateral connections [62]. The similar amplitudes of deviant and ‘many-standards' early responses in barrel cortex [61] are better explained by adaptation of intracortical synapses, as in our model, rather than adaptation in the thalamocortical synapses only [4], since the latter mechanism predicts higher responses in the ‘many-standards’ condition (see above for comparison of these two types of models).

In visual cortex, studies in cats [63] and monkeys [64] have found that complex cells adapt to a specific orientation while generally retaining their responses to other orientations. Such cells, therefore, show SSA to orientation. In contrast with auditory and somatosensory cortices, in visual cortex this phenomenon can be explained by a purely feedforward mechanism, with adaptation in simple cells (e.g. due to fatigue) shaping the tuning curves of complex cells in a stimulus-specific way (as suggested in [64]). Such a mechanism would be in line with the “cascading” adaptation characteristic of the visual system, e.g. in the way spatial adaptation in V1 appears to arise from integration of LGN responses [65] and motion adaptation in MT can be explained by broadly-tuned adaptation of their input V1 neurons [66,67]. Recently, true deviance sensitivity has been demonstrated in visual cortex using both extracellular recordings and calcium imaging, but the difference between deviant and many-standards responses occurs late [68]. This difference may challenge the purely feedforward explanation of adaptation in visual cortex, even if only indicating a late top-down modulation. We note that consideration of recurrent connectivity as essential to explaining V1 phenomena was emphasized in recent modeling work by Chariker et al. [69], although not in the context of adaptation.

### Generality of the model

Importantly, the mechanism generating SSA in our model generalizes to any stimulus or modality, if the tonotopic columns are replaced with populations coding for different stimuli or features. These populations need not be spatially segregated, but should have (i) stronger intra- vs. inter-population connection strengths and (ii) different sensitivities to afferent inputs. Thus, activity-dependent synaptic depression combined with heterogeneity in synaptic strengths leads to SSA for any stimuli encoded by the connectivity. In higher order areas of the auditory system, this can lead to SSA for complex stimuli.

## Materials and methods

### Modeling the input layer of A1

We model primary auditory cortex (A1), using multiple cortical columns (Fig 1A). Our model (described in detail below) closely follows Loebel et al. [28], which was based on the rate model of Wilson and Cowan [70] except that the basic units represented single neurons rather than local populations of neurons. A previous work [36] showed the equivalence of this rate model and a network of integrate & fire units in terms of the spontaneous and evoked activities, and importantly in the generation of population spikes. Our main modifications to the model of Loebel et al. [28] were the introduction of (i) synaptic depression in thalamocortical synapses and (ii) heterogeneity of tuning curves within each column, as suggested by imaging studies of mouse A1 [46,47]. We note that although cortical SSA was mostly reported in the cat [1] and rat [2,4,12,16], the physiological properties of A1, e.g. the heterogeneity of tuning curves on which we rely here, are better described in mice. All simulations, data analysis and calculations were performed using MATLAB (MathWorks).

Each column in our model is a fully-connected recurrent network: all neurons within the column are connected to all others. Our simulations show that population spikes occur also in a network with connection probabilities as found *in vitro* [27]. In general, even with a connection probability of around 0.1, as reported by Levy and Reyes [27] for connections between pyramidal neurons, the shortest path between any two neurons is on average less than 2 synapses (see e.g. [71]), and recruitment of the neuronal population into the population spike is rapid. Each column represents an iso-frequency band along the tonotopic axis of A1, covering about 1/3 of an octave, and consisting of *N*_*E*_ excitatory and *N*_*I*_ inhibitory neurons. Each neuron is described by two dynamic variables: firing-rate, denoted by *E*_*i*_^*Q*^ for excitatory and *I*_*l*_^*Q*^ for inhibitory neurons (*Q* specifies the column index and *i* or *l* the index of the neuron within the column); and the amount of resources available at each of the synapses made by this neuron, relative to its full resources, a fraction between 0 and 1 denoted *x*_*i*_^*Q*^ for excitatory and *y*_*l*_^*Q*^ for inhibitory neurons. Every neuron in the network receives external input, denoted *e*_*i*_^*E*,*Q*^ for excitatory and *e*_*l*_^*I*,*Q*^ for inhibitory neurons. External inputs are uniformly distributed within a specific range of firing-rates, i.e. each neuron receives input that depends linearly on its index within the column. All neurons within each column receive input from the excitatory populations of the nearest- and second-nearest neighboring columns, with connection strengths that decrease according to inter-column distance. Most excitatory neurons in the network also receive sensory input through a set of thalamocortical (ThC) synapses, each mediating a different tone frequency *f* and having its own dynamic variable, *z*_*i*_^*Q*,*f*^, representing the fraction of synaptic resources available at this synapse.

The network dynamics are defined by the following equations, following Loebel et al. [28] with the modifications described above:

$$\tau_{E}\frac{dE_{i}^{Q}}{dt}=-E_{i}^{Q}+\left(1-\tau_{ref}^{E}E_{i}^{Q}\right)\cdot g\left(\sum_{R=-2}^{2}\frac{J_{EE}^{\left|R\right|}}{N_{E}}\sum_{j=1}^{N_{E}}Ux_{j}^{Q+R}E_{i}^{Q+R}+\frac{J_{EI}}{N_{I}}\sum_{j=1}^{N_{I}}Uy_{j}^{Q}I_{j}^{Q}+e_{i}^{E,Q}+\sum_{f=1}^{M}U_{s}z_{i}^{Q,f}s_{f}T_{i}^{Q,f}\right)$$ (1)

$$\tau_{I}\frac{dI_{l}^{Q}}{dt}=-I_{l}^{Q}+\left(1-\tau_{ref}^{I}I_{l}^{Q}\right)\cdot g\left(\sum_{R=-2}^{2}\frac{J_{IE}^{\left|R\right|}}{N_{E}}\sum_{j=1}^{N_{E}}E_{j}^{Q+R}+\frac{J_{II}}{N_{I}}\sum_{j=1}^{N_{I}}I_{j}^{Q}+e_{l}^{I,Q}\right)$$ (2)

$$\frac{dx_{i}^{Q}}{dt}=\frac{1-x_{i}^{Q}}{\tau_{rec}^{E}}-Ux_{i}^{Q}E_{i}^{Q}$$ (3)

$$\frac{dy_{l}^{Q}}{dt}=\frac{1-y_{l}^{Q}}{\tau_{rec}^{I}}-Uy_{l}^{Q}I_{l}^{Q}$$ (4)

$$\frac{dz_{i}^{Q,f}}{dt}=\frac{1-z_{i}^{Q,f}}{\tau_{rec}^{s}}-U_{s}z_{i}^{Q,f}s_{f}T_{i}^{Q,f}$$ (5)

The units of our rate equations (Eqs 1 and 2) and rate variables (*E*, *I*, *e*, *s*) are 1/s. In the text and figures we specify the rate as Spikes/s for clarity.

*J* represents synaptic efficacy and has values depending on the type of connection and the distance over which it is made: *EE* denotes an excitatory-to-excitatory connection, *IE* an excitatory-to-inhibitory connection, *II* an inhibitory-to-inhibitory connection and *EI* an inhibitory-to-excitatory connection; the distance of the connection, in columns, is specified by a superscript *R* (*R* = 0 represents intra-columnar connections). *U* represents the fraction of utilization, i.e. how much of the available synaptic resources is utilized upon an action potential reaching the presynaptic terminal. *U*_s_ is the fraction of utilization of the ThC synapses. *τ*_*E*_ and *τ*_*I*_ are the membrane time-constants of excitatory and inhibitory neurons, respectively. *τ*_*ref*_ is the refractory period of excitatory or inhibitory neurons, as specified by the superscript *E* or *I*. *τ*_rec_ represents the time-constant for recovery of available synaptic resources in excitatory, inhibitory or ThC synapses, as specified by the superscript (*E*, *I* or *s*, respectively).

Eqs (1) and (2) describe the dynamical behavior of the excitatory and inhibitory neurons, respectively. These are rate equations–they describe the output rates of the neurons as a non-linear gain function of their summed inputs. The excitatory neurons receive intracortical excitation and inhibition (first two terms of the right-hand side of Eq 1) as well as direct excitatory thalamic input (the last term of the right-hand side of Eq 1). For simplicity, inhibitory neurons receive only intracortical excitation and inhibition, with no direct thalamocortical input. The lack of direct thalamo-cortical input to the inhibitory neurons in the model is discussed in length and justified in the main paper.

Most importantly for the special behavior of this model, the synapses impinging on excitatory neurons show depression. The model of synaptic depression implemented here consists of resource depletion followed by exponential recovery (Eqs 3–5). Each input class on the excitatory neurons has its own separate dynamical process of resource depletion and recovery.

The gain function used in the firing-rate equations is defined as:

$$g\left(w\right)=\{\begin{array}{cc} 0 & w<0\\ w & 0\leq w<E_{\max}\\ E_{\max} & w\geq E_{\max} \end{array}$$ (6)

Where *w* is the sum of inputs to a neuron, as detailed in Eqs 1 & 2. In all of our simulations, *Ε*_*max*_ was set to 300 spikes/s. The implementation of a maximum firing-rate follows [28]. This value for *Ε*_*max*_ is close to the effective maximum firing-rate that is set by the refractory period (*τ*_*ref*_) when its value is set to 3 ms (as we do here for both excitatory and inhibitory neurons; see list of values below).

Our model neurons sample the frequency axis in channels (*f*) that are ordered from 1 to *M*. In almost all types of protocols, *M* was equal to the number of columns and so *f* corresponds to *Q* (i.e. there is one channel per cortical column). The exceptions to this were the Diverse Narrow protocol, which required denser frequency channels, and the tests for hyperacuity (see below and in the Results section).

The input mediated by each ThC fiber is represented by a firing-rate variable *s*_*f*_. Its magnitude at each time-point depends on the maximum amplitude of the stimulus (*A*, measured in spikes/s) and the temporal envelope of stimuli presented in that channel (*ξ*_*f*_(*t*), a fraction between 0 and 1):

$$s_{f}=\xi_{f}\left(t\right)\cdot A$$ (7)

In all the stimuli presented in this work, *ξ*_*f*_(*t*) had the form of a trapezoid pulse: a 5-ms ascending linear ramp from 0 to 1, a period of constant amplitude, and a 5-ms linear ramp descending back to 0, all adding up to the nominal stimulus duration.

*T*_*i*_^*Q*,*f*^ specifies the relative amplitude at which an excitatory neuron receives input from frequency channel *f* compared to its best frequency (BF). The values of *T*_*i*_^*Q*,*f*^ over all channels make up the tuning curve of the neuron’s thalamic input. Each neuron was assigned a triangular-shaped input tuning curve. This shape was chosen in order to avoid having input to all columns upon each sensory stimulation, as was the case with the tuning curves used by Loebel et al. [28] that decayed exponentially from their peak at the best frequency. The width of the tuning curve is determined by a localization parameter *λ*, which scales the tuning curve according to the frequency separation between the columns. The equation for the input tuning curve of a typical neuron of column *Q*, i.e. with best frequency *f*_*BF*_ = *Q*, is:

$$T_{typical}^{Q,f}=\left\{ \begin{array}{cc} 0 & f<Q-\lambda \\ 1-\left(Q-f\right)/\lambda & Q-\lambda \leq f<Q\\ 1-\left(f-Q\right)/\lambda & Q\leq f<Q+\lambda \\ 0 & f\geq Q+\lambda \end{array}\right.$$ (8)

The value of *λ* = 5 used in our simulations confers upon the neurons in our model tuning widths of about 2 octaves (cf. Fig 1D), similar to the tuning of suprathreshold activity in rat A1 [40].

Recently, it has been shown that neurons within a local population of mouse A1 have heterogeneous best frequencies [46,47]. In line with these results, each column in our model contained neurons with several different best frequencies: Approximately 1/16, 1/8, 1/8 and 1/16 of the neurons in each column had their tuning-curves shifted by -2, -1, +1 and +2, respectively, relative to the typical *f*_*BF*_ = *Q*. Neurons with shifted input tuning-curves were selected randomly (Fig 1A illustrates the resulting tonotopy; the distribution is adjusted for illustration purposes). The ThC input to all neurons that had a firing-rate of 0 spikes/s when there was no sound input (“non-active” neurons) was zero, as proposed by Loebel et al. [28]. Calcium imaging studies in mice [47,72] suggest that the heterogeneity of tuning-curves implemented in our model is intermediate between those of layer 4 and layers 2/3 in mouse A1. We note that the randomization of tuning-curves was not done in order to generate independent samples, but in order to better model the physiology. We do not compare between networks with different randomized tuning-curves. Rather, almost all of our analyses were made on column averages. Our simulations of networks with homogeneous vs. heterogeneous columns showed that heterogeneity of the tuning curves slightly extended the parameter range that allowed stimulus-specific adaptation, but its effect was very small (cf. Fig 7B).

All simulations of this model used networks with 21 columns. The values we used for the different parameters were:

$$\begin{array}{l} N_{E},N_{I}=100;\;\lambda =5;\;U=0.5;\;U_{s}=0.7\\ \tau_{E},\tau_{I}=1\times 10^{-3}\;s;\;\tau_{ref}^{E},\tau_{ref}^{I}=3\times 10^{-3}\;s;\;\tau_{rec}^{E},\tau_{rec}^{I}=0.8\;s;\;\tau_{rec}^{s}=0.3\;s\\ J_{EE}^{0}=6;\;J_{IE}^{0}=0.5;\;J_{EI}=-4;\;J_{II}=-0.5;\\ J_{EE}^{1}=4.5\times 10^{-2};\;J_{IE}^{1}=3.5\times 10^{-3};\;J_{EE}^{2}=1.5\times 10^{-2},\;J_{IE}^{2}=1.5\times 10^{-3} \end{array}$$

Values of *e* for both populations were distributed uniformly between -10 and 10 spikes/s. The recovery time constants for both excitatory and inhibitory neurons are referred to as *τ*_rec_, since their values were identical in all of our simulations.

The values above are the same as in Loebel et al. [28]. Some were based on experimental studies, while others were tuned to obtain approximate balance of excitation and inhibition, a spontaneous firing-rate of a few spikes/s, and no occurrence of spontaneous PSs. All simulations were run using a time step *dt* = 1 x 10^−4^ s.

### Stimulus protocols

Stimuli are called “tones” here because they were set to simulate pure-tone stimulation. Each was presented in one frequency channel *f*. Since the behavior of the model in response to sequences of stimuli was of major interest here, tones were presented in sequences consisting of 100 stimuli. The number of different tones was determined by the protocol type [4,12]. In the oddball protocol there were 2 tones, the lower at frequency f1 and the higher at frequency f2, with a frequency separation Δf = f2 –f1. These tones were centered on the best frequency of the middle column of the network (column 11), using Δf = 2 unless specified otherwise. The deviant tone had a probability of occurrence P (P = 10% unless specified otherwise). The other tone, called the standard, occurred at a probability of 1 – P. Tone sequences were generated by a random permutation of a block of standard tones followed by a block of deviant tones, in a way that allowed deviants to occur close apart, or even one after another. Responses to the two conditions were calculated as averages over all occurrences, including deviant responses that were weaker due to recent deviants (cf. Fig 6B). This randomization is in line with the practice in experimental studies by our group. We do not treat the short-term effects of deviants on one another. For experimental results of this effect, see [43,73]. The oddball protocol was always run twice, first with f2 as deviant and f1 as standard and then with their roles reversed (Deviant f2 and Deviant f1; Fig 3F). The Equal protocol was similar to the oddball but had P = 50%. In the Deviant Alone protocol, all presentations of the standard tone were replaced with silent trials. We also used two sequences of “deviant-among-many-standards”, in which we presented 10 tones spaced evenly along the frequency scale (including f1 and f2), each occurring at a probability of 10%. The Diverse Narrow protocol had two tones lower than f1, four between f1 and f2, and two higher than f2. This required a denser sampling of the frequency axis, as the spacing between tones had to be Δf/5 (cf. Fig 4A). In the Diverse Broad protocol we used 10 tones with a spacing of Δf = 2, which is the same as in the oddball protocol. Four tones were below f1 and four were above f2 (cf. Fig 4A).

Individual stimuli had a duration of 50 ms, including onset and offset linear ramps of 5 ms. Inter-stimulus interval (ISI) was defined as the time between the onsets of consecutive stimuli. We used ISI = 350 ms, *A* = 5 spikes/s unless specified otherwise.

### Data analysis

Responses to stimuli were generally evaluated based on the mean firing-rate in the middle column, *E*, unless explicitly stated otherwise. In Figs 1C, 3B, 3D, 4G, 8B, 9C, 10A and 10B we present and analyze responses of other columns as well. For each stimulus, a spike-count was calculated by correcting *E* to the baseline (defined as the average of *E* during the 5 ms before stimulus onset) and then integrating it from stimulus onset to 45 ms after offset. Since this is a firing-rate model that has no sources of noise, a period as short as 5 ms is valid as a baseline. Its advantage is that it allowed us to calculate of the response over 45 ms even for the shortest ISIs used. The response to each tone was defined as the average spike-count for all presentations of that tone. In experimental studies of SSA the baseline is usually not subtracted (but see [12]). However, it should have little effect over the response sizes and strength of the SSA, since in general our simulations showed low spontaneous rates and very large responses.

We quantified the strength of SSA in a way that is becoming standard [1,2,4,8,9,12,16,60,61]. The responses in both conditions of the oddball protocol (Deviant f2, Deviant f1) were calculated and used to compute the Common-Contrast SSA Index (CSI):

$$\text{CSI}=\frac{d\left(\text{f}1\right)+d\left(\text{f}2\right)-s\left(\text{f}1\right)-s\left(\text{f}2\right)}{d\left(\text{f}1\right)+d\left(\text{f}2\right)+s\left(\text{f}1\right)+s\left(\text{f}2\right)}$$ (9)

Where *d*(f1) and *s*(f1) (*d*(f2) and *s*(f2)) are the responses to f1 (f2) when it was used as deviant and standard, respectively.

### Analytical treatment of feedforward synaptic depression

The dynamics of resources in each thalamocortical synapse (Eq 5) is a one-dimensional system, independent of the other variables in the network. The amount of resources at stimulus onsets or offsets during a train of standard stimuli can be treated as an iterated map, given that the stimulus duration and inter-stimulus intervals are constant throughout the stimulus protocol. For simplicity, we rewrite Eq 5 as:

$$\frac{dz_{i}^{Q,f}}{dt}=\frac{1}{\tau_{rec}^{s}}-\left(\frac{1}{\tau_{rec}^{s}}+U_{s}s_{f}T_{i}^{Q,f}\right)z_{i}^{Q,f}$$ (10)

When no stimulus is presented, the solution of this equation is:

$$z_{i}^{Q,f}\left(t\right)=1-\left[1-z_{i}^{Q,f}\left(t_{offset_{}}\right)\right]\exp\left(-\frac{t-t_{offset}}{\tau_{rec}^{s}}\right)$$ (11)

Where *t*_*offset*_ is the time when the last stimulus ended. For the period before the first stimulus in a protocol, we assume the system is in its steady-state and therefore *z*_*i*_^*Q*,*f*^ = 1 for all synapses. Under stimulus presentation, the solution of Eq 10 is:

$$z_{i}^{Q,f}\left(t\right)=\frac{1}{1+\tau_{rec}^{s}U_{s}s_{f}T_{i}^{Q,f}}-\left[z_{i}^{Q,f}\left(t_{onset}\right)-\frac{1}{1+\tau_{rec}^{s}U_{s}s_{f}T_{i}^{Q,f}}\right]\exp\left[-\left(1+\tau_{rec}^{s}U_{s}s_{f}T_{i}^{Q,f}\right)\frac{t-t_{onset}}{\tau_{rec}^{s}}\right]$$ (12)

Where *t*_*onset*_ is the onset of the current stimulus. Since we are interested only in the fractions of synaptic resources at stimulus onsets or offsets, we substitute the relevant time periods and get the following relations between the values of z for consecutive onset and offset times:

$$z_{onset}=1-k_{isi}\left(1-z_{offset}\right)$$ (13)

$$z_{offset}={\tilde{z}}_{ss}-k_{dur}\left(z_{onset}-{\tilde{z}}_{ss}\right)$$ (14)

Where:

$$\begin{array}{l} z_{onset}\equiv z_{i}^{Q,f}\left(t_{onset}\right);\;z_{offset}\equiv z_{i}^{Q,f}\left(t_{offset}\right);\;{\tilde{z}}_{ss}\equiv \frac{1}{1+\tau_{rec}^{s}U_{s}s_{f}T_{i}^{Q,f}};\\ k_{isi}\equiv \exp\left(-\frac{t_{onset}-t_{offset}}{\tau_{rec}^{s}}\right);\;k_{dur}\equiv \exp\left(-\frac{t_{offset}-t_{onset}}{\tau_{rec}^{s}{\tilde{z}}_{ss}}\right) \end{array}$$

Substituting Eq 13 in Eq 14, and vice versa, gives the two iterated maps:

$$z_{onset}\left(n+1\right)=1-k_{isi}\left[1-{\tilde{z}}_{ss}\left(1-k_{dur}\right)\right]+k_{dur}k_{isi}z_{onset}\left(n\right)$$ (15)

$$z_{offset}\left(n+1\right)={\tilde{z}}_{ss}\left(1-k_{dur}\right)+k_{dur}\left(1-k_{isi}\right)+k_{dur}k_{isi}z_{offset}\left(n\right)$$ (16)

Which have the joint stable fixed points:

$$z^{*}{}_{onset}=\frac{1-k_{isi}\left[1-{\tilde{z}}_{ss}\left(1-k_{dur}\right)\right]}{1-k_{dur}k_{isi}}$$ (17)

$$z^{*}{}_{offset}=\frac{{\tilde{z}}_{ss}\left(1-k_{dur}\right)+k_{dur}\left(1-k_{isi}\right)}{1-k_{dur}k_{isi}}$$ (18)

Eqs 17 and 18 are the expressions for the onset and offset values of *z* reached after enough identical stimuli were presented. These values were used in Fig 9C and 9D, as the approximate steady-state resources in synapses conveying the standard stimuli from the thalamus to the standard column (the column whose best frequency is the standard stimulus). They are correct when all neurons in the column have the same best frequency, when the stimulus sequence consists of standard tones only, and when stimulation is on-off only (no ramp in the stimulus envelope, i.e. *ξ*_*f*_(*t*) consists only of square pulses).

The MATLAB scripts for running our model and the data replotted from [4,12,45] are available online as supporting information.

## Supporting information

S1 Code Matlab code for initializing the model network. (M)

S2 Code Matlab code for running the model network. (M)

S1 Data Fig 3 replotted data. (XLSX)

S2 Data Fig 4 replotted data. (XLSX)

S3 Data Fig 5 replotted data. (XLSX)
